# Supplementary material for: The genome sequence of the Provence Hairstreak, Tomares ballus (Fabricius, 1787) (Lepidoptera: Lycaenidae)
Source: Wellcome Open Res. 2026 Jan 16;11:35. [Version 1] doi: 10.12688/wellcomeopenres.25725.1 (PMC12910201; doi:10.12688/wellcomeopenres.25725.1)
Supplement: Peer review history

# Reviewer report: Reviewer response for version 1

**DOI:** 10.21956/wellcomeopenres.28323.r146206

**Published:** 2026-02-16

**Authors:** Noah Rose

**Affiliations:** 1 University of California San Diego, San Diego, USA

The authors present the genome of the Provence Hairstreak. The methods are state of the art and the resulting assembly is of good quality -- chromosome scale with resolved haplotypes, with a mitochondrial assembly too. The species is the sole representative of the tribe Tomarini, so the genome will have considerable value for comparative genomics and conservation genomics work.

Are sufficient details of methods and materials provided to allow replication by others?

Yes

Is the rationale for creating the dataset(s) clearly described?

Yes

Are the datasets clearly presented in a useable and accessible format?

Yes

Are the protocols appropriate and is the work technically sound?

Yes

Reviewer Expertise:

Ecological genomics

I confirm that I have read this submission and believe that I have an appropriate level of expertise to confirm that it is of an acceptable scientific standard.

# Reviewer report: Reviewer response for version 1

**DOI:** 10.21956/wellcomeopenres.28323.r146201

**Published:** 2026-02-13

**Authors:** Peng-Fe Ma

**Affiliations:** 1 Kunming Institute of Botany, Chinese Academy of Sciences, Kunming, China

In this study,  the high-quality genome assembly of Tomares ballus was reported, the genus of which is the sole representative of the tribe Tomarini within the Theclinae subfamily. The assembly contains two haplotypes with one of them scaffolded into the chromosomal level at 23  chromosomes. Analyses of K-mer and BUSCO both indicate a high completeness for the genome assembly. In short. the genomic resource generated will be valuable for studying the evolution of butterflies in future.

Are sufficient details of methods and materials provided to allow replication by others?

Yes

Is the rationale for creating the dataset(s) clearly described?

Yes

Are the datasets clearly presented in a useable and accessible format?

Yes

Are the protocols appropriate and is the work technically sound?

Yes

Reviewer Expertise:

Genomics, plant evolution.

I confirm that I have read this submission and believe that I have an appropriate level of expertise to confirm that it is of an acceptable scientific standard.
